# Development of an Immortalized Porcine Fibroblast Cell Panel With Different Swine Leukocyte Antigen Genotypes

**DOI:** 10.3389/fgene.2022.815328

**Published:** 2022-02-07

**Authors:** Quy Van Chanh Le, SeungYeon Youk, Munjeong Choi, Hyoim Jeon, Won-Il Kim, Chak-Sum Ho, Chankyu Park

**Affiliations:** ^1^ Department of Stem Cell and Regenerative Biotechnology, Konkuk University, Seoul, South Korea; ^2^ College of Veterinary Medicine, Chonbuk National University, Iksan, South Korea; ^3^ Gift of Hope Organ & Tissue Donor Network, Itasca, IL, United States

**Keywords:** fibroblast, immortalization, swine leukocyte, pig, major histocompatibility complex, hTERT

## Abstract

Immortalized cell lines are valuable resources to expand the molecular characterization of major histocompatibility complex genes and their presented antigens. We generated a panel of immortalized cell lines by transfecting human telomerase reverse transcriptase (*hTERT*) into primary fibroblast cells prepared from ear, fetal, and lung tissues of 10 pigs from five breeds and successfully cultured them for 30–45 passages. The cell growth characteristic of the immortalized fibroblasts was similar to that of primary fibroblast, which was unable to form colonies on soft agar. The genotypes of major swine leukocyte antigen (SLA) genes, including three classical class I (*SLA-1, -2,* and *-3*) and three class II genes (*DQB1*, *DRB1*, and *DQA*), were determined using high-resolution typing. A total of 58 alleles, including a novel allele for *SLA-2*, were identified. Each cell line was unique. A cell line derived from a National Institutes of Health miniature pig was homozygous across the six major SLA genes. The expression levels of SLA classical class I genes varied among the cell lines and were slightly upregulated in the immortalized compared to the primary cells based on semiquantitative reverse transcription polymerase chain reaction. The immortalized porcine fibroblast cell lines with diverse SLA haplotypes that were developed in this study have potential to be applied in studies regarding the molecular characteristics and genetic structure of SLA genes and epitope–major histocompatibility complex interactions in pigs.

## Introduction

Antigen presentation by major histocompatibility complex (MHC) class I and II molecules is an essential process in adaptive immune interactions that triggers T cell proliferation and antigen-specific cellular immune responses (Wieczorek et al., 2017). MHC class I molecules are expressed on the surface of all nucleated cells and interact with cytotoxic T cells (CTL) to trigger immune responses against intracellular pathogenic peptides, derived from viral proteins and tumor cells (Kaufman et al., 1990; Sommer, 2005). In contrast, MHC class II molecules are expressed only by professional antigen-presenting cells, including dendritic cells, mononuclear phagocytes, B cells, among others, and peptide/MHC class II complexes interact with T helper cells to mediate immune responses against extracellular pathogens (Lechler et al., 1991; Ting and Trowsdale, 2002). Therefore, the engagement of pathogen-derived antigenic peptides with MHC molecules, before recognition by surveillance T lymphocytes, plays a critical role in adaptive immunity.

MHC genes are extremely polymorphic and generating distinctive structural variations, especially in the antigen binding pockets that interact with antigenic peptides by anchoring amino acids (Rammensee, 1995). A single MHC molecule can bind to multiple peptides and a peptide could be presented by several different MHC molecules (Van Hateren et al., 2010; Eisen et al., 2012). This increases the potential diversity of antigenic peptides repertoire presented to T cells from endogenous and exogenous environments.

The activation of primary T cells requires the integration of three distinct signals, namely the three-signal theory of activation including antigen recognition, costimulation, and cytokine-mediated differentiation and expression (Sckisel et al., 2015). In these sequential processes, only antigenic peptides with stable and strong bonds to MHC molecules can be presented to T cells and induce antigen-specific immune responses (Wooldridge et al., 2012). Therefore, a detailed understanding of binding kinetics between MHC molecules and antigenic peptides is necessary to improve the efficiency of epitope screening in the development of new generation vaccines and accurate predictive algorithms (Stienekemeier et al., 2001; Lazoura and Apostolopoulos, 2005; Reche et al., 2006). In addition, the determination of MHC alleles strongly interacting with antigenic peptides of major pathogens could be exploited as a strategy to increase the resistance of animals by selecting individuals with resistance haplotypes in population (Bosch-Camós et al., 2021).

Determination of MHC alleles is important to predict adaptive immune responses of an individual. The genomic sequence-based high-resolution typing methods for SLA genes have been developed (Park et al., 2010; Thong et al., 2011; Le et al., 2012; Choi H. et al., 2015; Le et al., 2015). However, all available methods are limited to genotype a single locus. Thus, the development of high throughput typing methods suitable for parallel typing of multiple SLA genes are in need.

Several methods have been developed to estimate interaction kinetics between antigens and MHC molecules using cell-free or animal cell systems (Yaneva et al., 2010). Compared to artificial cell-free assays in bacteria and yeasts, the method using live animal cells does not require the cloning and expression of MHC or antigenic molecules. When screening against a large number of MHC alleles, the live animal cell system requires reduced labor investment for greater reproducibility (Townsend et al., 1990; Cerundolo et al., 1991; Elvin et al., 1991; Sette et al., 1994; Ottenhoff et al., 1997; Tan et al., 1997; Buus et al., 2001; Sidney et al., 2001; Narzi et al., 2008). The native structure of MHC molecules on the surface of live mammalian cells allows for a more representative characterization of antigen biding interactions *in vivo* compared to the cell-free system (Bodmer et al., 1989; Ljunggren et al., 1990; Storkus et al., 1993; Zeh et al., 1994; Bremers et al., 1995; van der Burg et al., 1995; Kessler et al., 2001; Turvy and Blum, 2001; Palankar et al., 2009).

Many cell lines with well characterized MHC genotypes are available for molecular characterization of human lymphocyte antigens (Sckisel et al., 2015) and epitope binding and elution assays (Townsend et al., 1990; Tan et al., 1997; Kessler et al., 2001). However, the availability of these cell lines is limited for livestock species, and only eight cell lines with genotype information of major SLA genes are available in pigs (Schook et al., 2005; Vodicka et al., 2005; Ho et al., 2009; Meurens et al., 2012). Also, the alleles of available cell lines often overlap, further limiting the coverage of analyses using major swine leukocyte antigens (SLA) alleles (Ho et al., 2009).

In immunopeptidome studies, the number of cells that are evaluated per experiment is one of the most important limitations (Melief and Kessler, 2017), and immortalization could solve that condition. The objective of this study was to establish and characterize a cell panel consisting of 10 immortalized fibroblast cell lines containing 58 SLA alleles. The availability of cell lines covering diverse alleles of SLA could serve as a valuable resource for immunogenetic analysis in pigs and the development of high-throughput SLA typing methods.

## Materials and Methods

### Tissues and Cell Preparation

Tissue samples (ear notch or lung tissue) were obtained from five purebred landraces on local farms, two NIH miniature pigs, one PWG micropig (PWG Genetics, Korea), and one Seoul National University miniature pig (SNU, Seoul, Korea) (Choi H. et al., 2015; Choi M. et al., 2015; Le et al., 2020). All animals were 9 weeks old. A Yorkshire uterus, with fetus, was obtained from a local slaughterhouse (Seoul, South Korea). PK15, a pig kidney fibroblast cell line (ATCC CCL-33), was purchased from American Type Culture Collection (ATCC, Manassas, VA, United States). Primary pig fibroblast cells were isolated as previously described (Denning et al., 2001). Briefly, tissue samples were rinsed in 1x phosphate buffered saline (PBS) solution containing 1,000 U/ml penicillin and 500 μg/ml streptomycin (Gibco, NY, United States), treated with 1x Antibiotic-Antimycotic (Gibco), minced with a surgical blade in a culture dish, and disaggregated in 0.25% Trypsin-EDTA (Gibco) at 37°C for 1 h in a 5% CO_2_ incubator (ASTEC, Fukuoka, Japan). The disaggregated cells were washed in PBS and cultured in six-well plates using Dulbecco’s Modified Eagle’s Medium (DMEM) (HyClone, UT, United States) with 10% fetal bovine serum (FBS) (Thermo Scientific, MA, United States) and 20 μg/ml gentamycin (Sigma-Aldrich, MO, United States). Subsequently, the cells were cultured in DMEM supplemented with 10% FBS and a 1% penicillin-streptomycin antibiotic mixture (Gibco). All experimental procedures were approved and performed accordance with the guidelines and regulations set by the Institute of Animal Care and Use Committee (IACUC) and the Center for Research Ethincs of Konkuk University (KU20229).

### High Resolution SLA Typing

Genomic DNA was extracted from ∼0.5 g of tissue using the standard protocol (Sambrook et al., 1989). Briefly, tissues were incubated with lysis buffer [10 mM Tris-HCl (pH 8.0) and 0.1 M EDTA] containing 0.5% sodium dodecyl sulfate and 1 mg/ml proteinase K (Promega, Madison, WI, United States) at 55°C for 6 h, followed by phenol extraction. For cells, DNA and RNA were isolated simultaneously from 5×10^6^ cells using an AllPrep DNA/RNA Mini Kit (Qiagen, Hilden, Germany) according to the manufacturer’s protocol. Genomic DNA-based high-resolution typing of *SLA-1, SLA-2, SLA-3, SLA-DQA, SLA-DQB1*, and *SLA-DRB1* was conducted for each gene as previously described (Park et al., 2010; Thong et al., 2011; Le et al., 2012; Choi H. et al., 2015; Le et al., 2015; Le et al., 2020). Briefly, locus-specific amplicons of each SLA gene were generated in a 20 μL reaction containing 50–100 ng of genomic DNA, 0.5 μM primers (SLA1-e1F1 and SLA-e4R4, SLA2-e1F2 and SLA-e4R4, SLA3-spF5 and SLA-e4R4, DQAi1F3 and DQAe3R1, DQB1F-119 and DQB1R+295, and DRB1F-22 and DRB1R+284 for *SLA-1, SLA-2, SLA-3, SLA-DQA, SLA-DQB1*, and *SLA-DRB1*, respectively, Supplementary Table S1), 200 μM dNTPs, 1x PCR buffer, and 0.5 U of Supertherm™ DNA polymerase (JMR Holdings, Kent, United Kingdom) using an ABI 9700 Thermocycler (Applied Biosystems, Foster City, CA, United States). The cycling profiles consisted of the following steps: initial denaturation at 95°C for 5 min, 35 cycles of denaturation at 95°C for 1 min, annealing at a specific temperature for each locus (Supplementary Table S1) for 1 min, extension at 72°C for 2 min, and a final extension at 72°C for 5 min. PCR products were analyzed by 1.5% agarose gel electrophoresis. Amplicons were then subjected to direct sequencing using locus-specific sequencing primers (SLA1-Seq. 2-F and SLA1-Seq. 3-F, SLA-i1F and SLA-i2F, SLA3-seq2-F6, SLA3-seq2-R7, SLA3-seq3-F4 and SLA3-seq3-R5, DQAi1F4, Sq-mul-DQB1 and Sq-mul-DRB1 for *SLA-1, SLA-2, SLA-3, SLA-DQA, SLA-DQB1*, and *SLA-DRB1*, respectively, Supplementary Table S1) using an ABI PRISM BigDye™ Terminator Cycle Sequencing Kit (Applied Biosystems) following the manufacturer’s protocol. Allelic determination of each SLA gene was conducted by aligning the sequence-based typing results with the annotated reference sequences from a local SLA database (containing all SLA sequences uploaded to GenBank) using a multiple alignment tool, CLC Main Workbench 7 (Qiagen CLC bio, Hilden, Germany). To separate and identify each allele in the heterozygotes, the PCR products were cloned when the pattern was observed for the first time, and then the individual clones of each allele were sequenced as described previously (Thong et al., 2011). At least two clones for each allele were sequenced from both the 3′ and 5′ directions. Observed heterozygosity was calculated as the number of heterozygotes divided by the total number of individuals.

### Transfection and Selection of Cells

Cells were transfected with *pBABE-hTERT-Puro* (Counter et al., 1998) using Lipofectamin^TM^ 3000 (Thermo Scientific) following the manufacturer’s protocol. Cell selection occurred after 48 h when transfected cells were placed under selective pressure by adding 2 μg/ml puromycin (Sigma-Aldrich, Germany) for 4 weeks. The cells were transferred and cultured in DMEM supplemented with 10% FBS and a 1% penicillin-streptomycin antibiotic mixture (Gibco) at 37°C and 5% CO_2_. Cells were frozen overnight at −80°C in DMEM containing 20% FBS and 10% dimethyl sulfoxide, without antibiotics, and then stored in liquid nitrogen. Before use, the cells were rapidly thawed in a water bath at 37°C.

### Characterization of Cell Growth and Soft Agar Assay

Cells (2  ×  10^5^ cells/well) were seeded in a six-well plate and cultured in the same conditions as described for cell selection. Cells were trypsinized at 24, 48, and 72 h post-seeding, counted using the trypan blue exclusion method (Strober, 2001), and the number of cells were plotted against each time point. To measure cell anchorage-independent proliferation, 1 × 10^4^ cells/well of randomly selected immortalized fibroblasts were cultured for 2 weeks in a six-well soft agar dish with 0.8% agar and 0.7% agarose in the bottom and upper layers, respectively. Cells were fed with cell culture media every 2 days. Colony formation was monitored using a light microscope (Model BX51 TF, Olympus, Tokyo, Japan).

### Expression Analysis of *hTERT*

Total RNA from 2 × 10^5^ cells was extracted using an RNA/DNA mini kit (Qiagen, Hilden, Germany) and subjected to cDNA synthesis by SuperiorScript III Reverse Transcriptase (Enzynomics, Korea) according to the manufacturer’s protocol. PCR was performed in a 20 µL reaction mixture containing 2 µL cDNA, 0.5 µM primers, 0.5 U of Supertherm DNA polymerase (JMR Holdings) in 1.2x PCR buffer (1.5 nM MgCl_2_), and 0.1 mM dNTPs using the ABI 9700 Thermocycler (Applied Biosystems). The amplification primers for *hTERT* and Glyceraldehyde-3-phosphate dehydrogenase (GAPDH) were hTERT-F (5′- GCC​GAG​ACC​AAG​CAC​TTC​CTC​TAC​T-3′) and hTERT-R (5′-GCA​ACT​TGC​TCC​AGA​CAC​TCT​TCC​G-3′), and GAPDH-SP-F (5′-GCT​ACA​CTG​AGG​ACC​AGG​TTG-3′) and GAPDH-SP-R (5′- AGG​AGA​TGC​TCG​GTG​TGT​TG-3′), respectively. The PCR profile consisted of an initial denaturation cycle of 5 min at 95°C, 35 cycles for 30 s at 95°C, 30 s at 63°C, 45 s at 72°C, and a final extension of 5 min at 72°C for *hTERT*. The cycling conditions for *GAPDH* was similar to that of *hTERT* except that the denaturation cycle was 3 min (at the same temperature), the number of annealing cycles was 25 at 58°C, and extension was performed for 30 s at 72°C. Subsequently, PCR products were electrophoresed on 1% agarose gel and band intensity was compared between samples.

### Expression Analysis of SLA Genes and *Vimentin*

Isolated total RNA was subjected to RNase-free DNaseI (Qiagen Sciences Inc., Germantown, MD, United States) treatment, and RNA quality was analyzed on 2% formaldehyde agarose gel. Reverse transcription was performed in a 20 μL reaction mixture using 1 μg of total RNA with oligo-(dT)15 and SuperScript® III Reverse Transcriptase (Invitrogen, MA, United States), for 50 min at 50°C with inactivation for 15 min at 72°C. In contrast to the materials used in SLA typing, the target cDNA was amplified using 2 μL of cDNA with Pyrobest polymerase (TaKaRa Bio, Shiga, Japan). The primers used to amplify the entire coding region of *SLA-1, SLA-2, SLA-3*, and *SLA-DRB1* were SLA1-e1F1 and SLA-e4R4, SLA2-e1F and SLA-e4R4, SLA1/3f#92 and SLA3r#121, CpDRB5a and CpDRB3b, respectively (Ho et al., 2006; Thong et al., 2011; Choi H. et al., 2015; Le et al., 2020) (Supplementary Table S1). The thermal cycling profile included a 3 min denaturation step at 95°C, 32 cycles of denaturation for 30 s at 95°C, annealing for 30 s at 65°C, 61°C, 50°C, and 63°C for SLA-1, -2, -3, and -DRB1, respectively, 5 min extension at 72°C, and a final extension of 30 min at 72 °C. The amplification of *vimentin* was carried out using a primer set, Vimentin-F and Vimentin-R (Supplementary Table S1, Shi et al., 2013), using 2 μL of cDNA in the same way as above under the PCR profile consisting of an initial denaturation cycle of 3 min at 95°C, 35 cycles for 30 s at 95°C, 30 s at 58°C, 30 s at 72°C, and a final extension of 5 min at 72°C. *GAPDH* was used as a control. The level of expression was estimated as the photo density ratio of the *SLA-1*, *-2*, *-3*, and *-DRB1* amplicons relative to *GAPDH*.

### SLA Class I Immuno-Cytochemical Analysis

Cells were seeded on glass cover slips in 6-well plates at a density of 1.2 × 10^5^ cells/well in DMEM in the same conditions described above. After 2°days, cover slips with attached cells were washed three times with PBS and fixed with 4% paraformaldehyde for 15 min at room temperature. Cells were washed three times with cold PBS before blocking with 5% bovine serum albumin (BSA) in PBS for 30 min at room temperature. The cells were incubated with a mouse monoclonal antibody specific to pig SLA Class I (Bio-Rad, CA, United States) at 1:250 dilution in PBS with 2.5% BSA at room temperature for 2 h. After washing in PBS, cells were incubated with goat anti-mouse antibody conjugated with Alexa Fluor 568 (Invitrogen) at a dilution of 1:500 in PBS containing 3% BSA for 1 h at room temperature in the dark. After washing in cold PBS, cover slips were mounted with Vectashield (Vector, Burlingame, CA, United States). The cells were observed under a fluorescence microscope (BX51TF, Olympus).

## Results

### Genetic Diversity of the Three SLA Classical Class I and Three Class II Genes

To establish a panel of cell lines with diverse SLA haplotypes, we conducted high-resolution typing of three SLA classical class I genes, *SLA-1*, *SLA-2*, *and SLA-3*, and three SLA class II genes, *SLA-DQA*, *SLA-DQB1*, and *SLA-DRB1*, from 10 individuals of five different breeds (Table 1). A total of 16, 13, 8, 6, 8, and 7 alleles were identified for *SLA-1*, *SLA-2*, *SLA-3*, *SLA-DQA*, *SLA-DQB1*, and *SLA-DRB1*, respectively. A new *SLA-2* allele was identified showing 27 nucleotide differences from SLA-2*13:02. The sequence of the new allele was confirmed by cloning, submitted to NCBI (accession number MZ700355), by the ISAG SLA nomenclature committee.

**TABLE 1:** High-resolution typing results of six SLA genes for 10 immortalized fibroblast cell lines.

Cell line	Passage	Breed	Tissue	*SLA-1*	*SLA-2*	*SLA-3*	*DQA*	*DQB1*	*DRB1*
iKUF01	40	PWG	Ear	13:01/11:04/12:03	04:02	04:01	02:04/05:01	03:03/05:02	04:03/07:01
iKUF02	35	Landrace	Lung	16:01/08:10	12:01/10:09	06:01	01:01	01:01/06:01	01:01/10:01
iKUF03	35	NIH	Ear	02:01/04:01/07:01	02:01/04:01	04:01/03:03	02:01	02:01/04:01:01	02:01
iKUF04	30	NIH	Ear	04:01	04:01	04:01	02:01	04:01:01	02:01
iKUF05	34	Landrace	Ear	11:02/15:02	16:01/15:01	03:03/07:01:02	02:01/02:04	02:01/03:03	02:01/04:03
iKUF06	30	Landrace	Ear	11:02/15:01/09:01	15:01/05:03	03:03/07:01:02	01:01/02:01	02:01/06:01	02:01/10:01
iKUF07	30	Landrace	Lung	07:02/14:01	02:02/10:04	03:03/05:01	01:06	07:01	06:02
iKUF08	32	Yorkshire	Fetus	04:01/21:01/11:03	04:02:01/16:03	04:01/03:06	01:06/02:01	02:01/07:01	02:01/06:02
iKUF09	42	Landrace	Ear	15:01/09:01	04:01/05:03	06:02/04:01	01:01/02:01	04:01:01/06:01	02:01/10:01
iKUF10	45	SNU	Ear	02:01/07:01	03:01/21:01	03:01	01:02	03:01	03:01

Passage numbers are indicated within parentheses together with the names of the cell lines.

The mean heterozygosity of *SLA-1*, *SLA-2*, *SLA-3*, *SLA-DQA*, *SLA-DQB1*, and *SLA-DRB1* was 90%, 80%, 60%, 50%, 70%, and 60%, respectively, with *SLA-1* and *DQA* showing the highest and the lowest heterozygosity, respectively*.* The typing results for individuals corresponding to iKUF cells 02, 04, 06, and 09 showed the presence of three alleles in *SLA-1*, indicating the presence of the duplication haplotypes. The allelic constitution for each SLA gene was consistent to the reported allelic frequencies of the corresponding breeds (Park et al., 2010; Thong et al., 2011; Le et al., 2012; Choi H. et al., 2015; Le et al., 2015; Le et al., 2020).

In our cell panel, iKUF04, which originated from an inbred NIH miniature pig, was homozygous across all six loci (*SLA-1*: 04:01; *SLA-2*: 04:01; *SLA-3*: 04:01; *DQA*: 02:01; *DQB1*: 04:01:01; *DRB1*: 02:01) (Table 1). Based on this, we were able to determine the haplotype of iKUF03 (*SLA-1*: 02:01-07:01; *SLA-2*: 02:01; *SLA-3*: 03:03; *DQA*: 02:01; *DQB1*: 02:01; *DRB1*: 02:01) by subtracting the identified haplotype of iKUF04 from the genotyping results of iKUF03. For the rest of the cell lines, we were unable to separate the combined genotyping results of the six SLA genes into individual haplotypes due to the unavailability of family typing results.

### Establishment of 10 Immortalized Porcine Fibroblast Cell Lines With High Resolution SLA Genotypes

**FIGURE 1:**
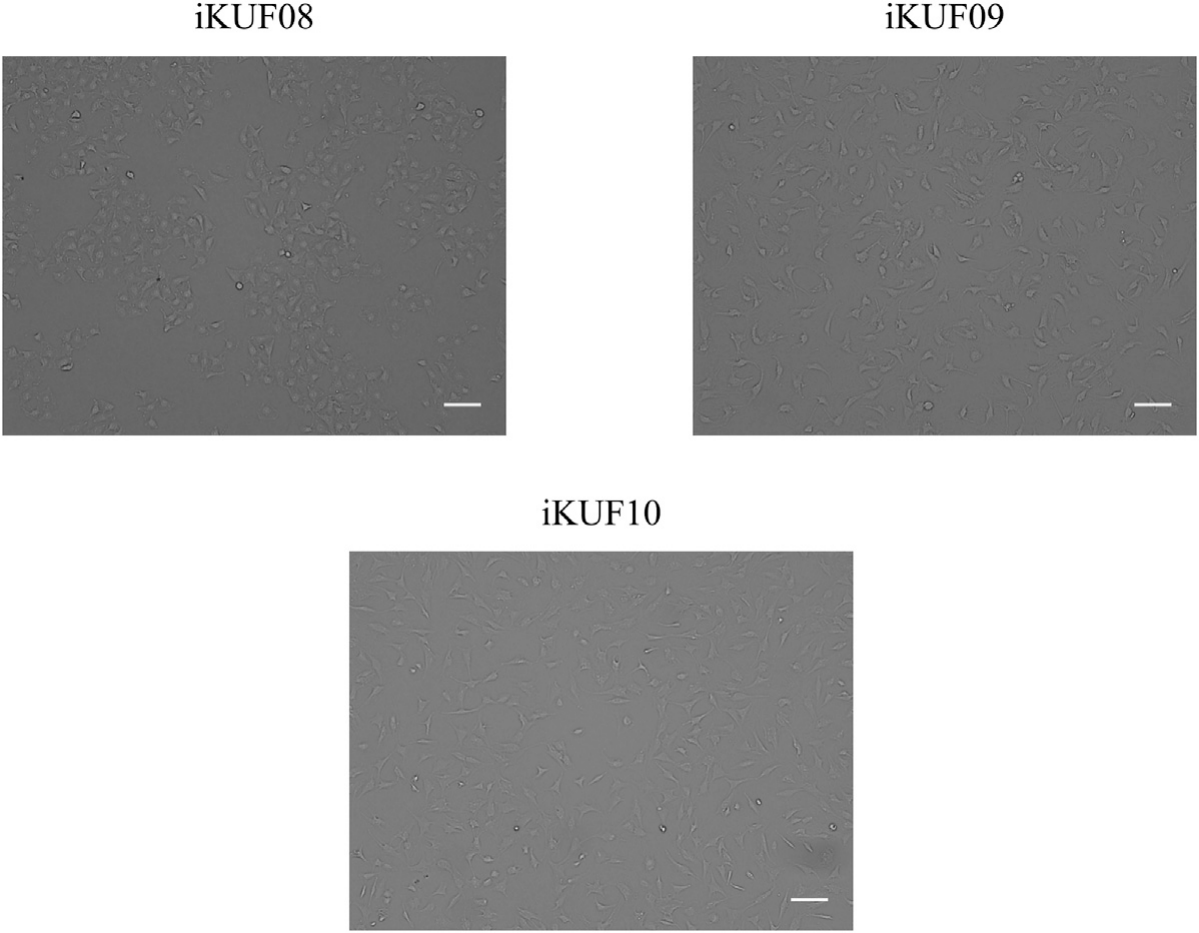
Morphological comparison of immortalized porcine fibroblast cell lines. Three cell lines, iKUF08, iKUF09, and iKUF10, were randomly selected from 10 developed cell lines. The names of the cell lines are indicated on top. Scale bar: 100 µm. Magnification: × 10.

**FIGURE 2:**
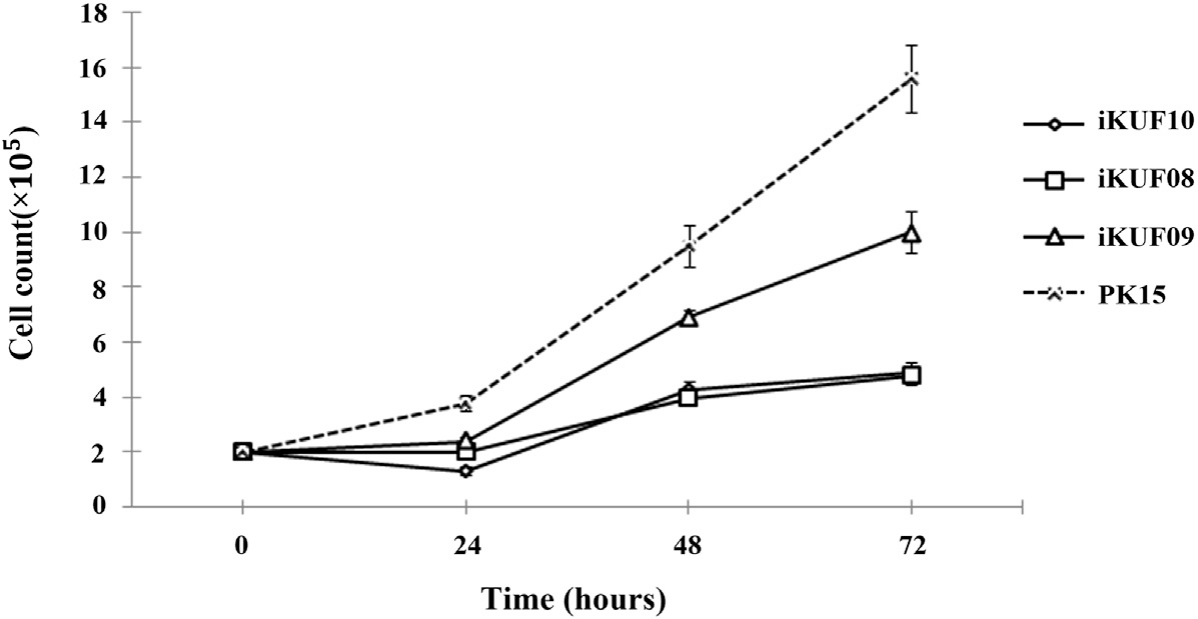
Growth curves of immortalized fibroblast cells. Three randomly selected cell lines, iKUF08, iKUF09, and iKUF10, and a commercially available PK15 were used. The culture duration is indicated on the x-axis.

Primary fibroblast cells obtained from different tissues of 10 pigs were transfected with the *hTERT* expression construct. After culturing for 4 weeks in the selection medium containing puromycin, ∼10–50% of cells survived. A small number of cells were found to grow rapidly from enlarged and flattened senescent cells when they were relaxed in a puromycin-free culture. These cells were isolated and cultured until passage number 20 to make a homogeneous population. The passage numbers of the established fibroblast cell lines ranged from 30 to 45 (Table 1). We considered the cells with uniform morphology after 30 passages to be immortalized (Figure 1). To characterize the growth of the immortalized cells, we randomly selected three cell lines, iKUF08, iKUF09, and iKUF10, and compared their proliferation rates together with that of PK15, a commercially available porcine fibroblast cell line (Figure 2). Cell proliferation rates were much higher in PK15 and iKUF09 than in iKUF08 and iKUF10, showing variations in cell growth among them. The difference in proliferation rates among iKUF08, iKUF09, and iKUF10 was not associated to passage number (Figure 2 and Table 1).

When the *hTERT* expression of immortalized cell lines was evaluated using semiquantitative reverse transcription PCR, the 778 bp *hTERT* specific amplicon was observed in all the cell lines (Supplementary Figure S1). The level of *hTERT* expression varied among the cell lines with iKUF01, iKUF05, iKUF08, iKUF09, and iKUF10 showing the highest levels of expression. The level of *hTERT* expression was not directly related to proliferation rate (Figure 2 and Supplementary Figure S1).

### Anchorage-independent Growth of Immortalized Fibroblast Cells

**FIGURE 3:**
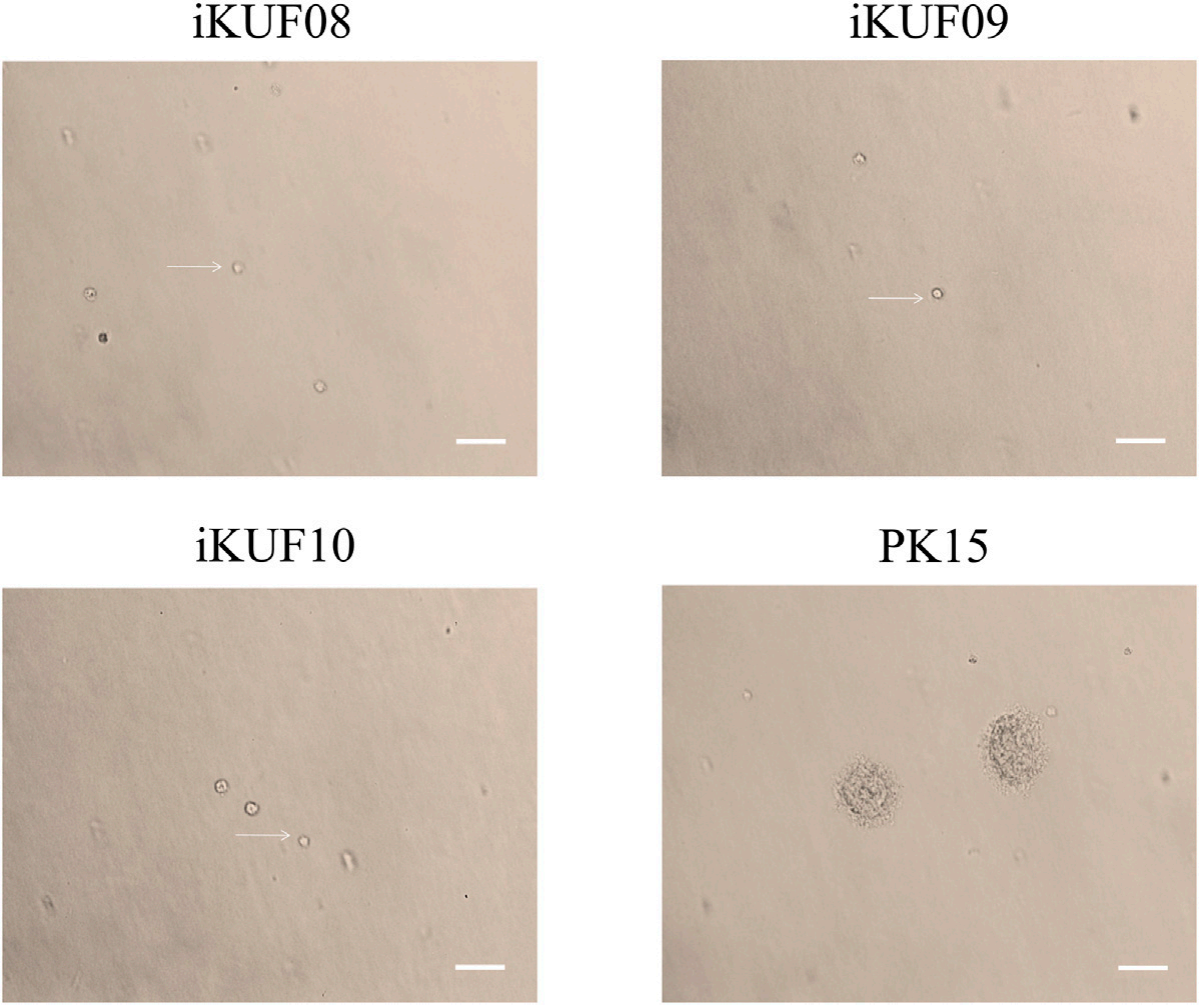
Anchorage-independent growth of immortalized porcine fibroblast cells in soft agar. Three randomly selected cell lines, iKUF08, iKUF09, and iKUF10, and a commercially available PK15 were used. The cell names are indicated on top. Arrows indicate single cells. Scale bar: 100 µm. Magnification: × 10.

Anchorage-independent growth is the ability of transformed cells to grow without requiring a solid surface. We analyzed the anchorage dependency of cell growth for PK15 as well as three randomly selected cell lines, iKUF08, iKUF09, and iKUF10 (with passage numbers 32, 42, and 45, respectively). All four cell lines grew well on soft agar, with PK15 forming numerous colonies. In contrast, the *hTERT* transfected cells did not form any colonies, even after 2 weeks of culture (Figure 3).

### Variations of SLA Classical Class I Gene Expression Among Immortalized Fibroblast Cell Lines

**FIGURE 4:**
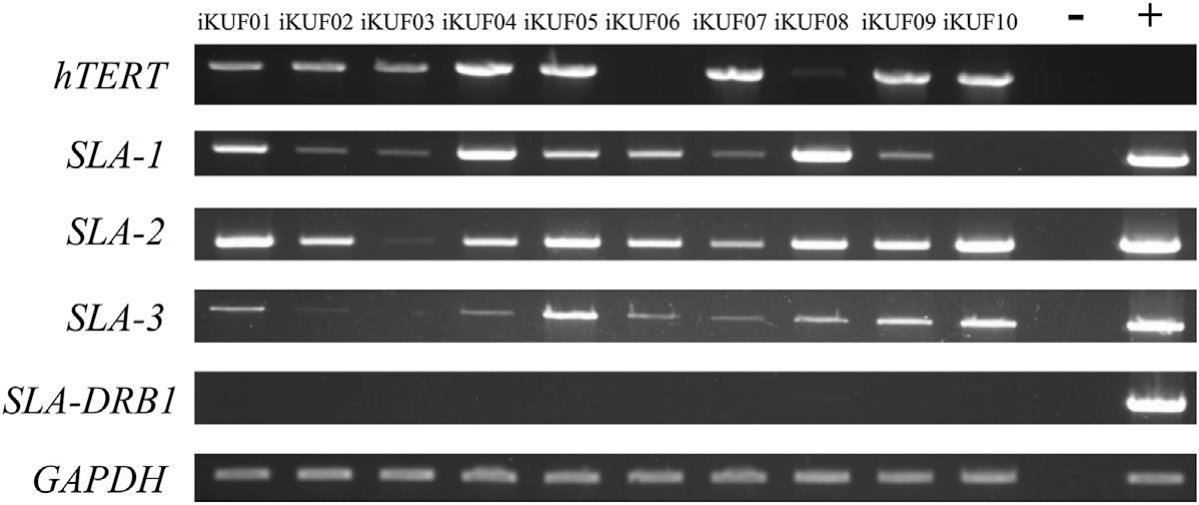
Comparison of the expression of *SLA-1, -2, -3*, and *-DRB1* in immortalized fibroblast cell lines. Semi quantitative reverse transcription PCRs were carried out for *SLA-1, -2, -3*, and *-DRB1*. *GAPDH* was used as a control. The numbers of the cell lines are on top. “–” and “+” indicate negative and positive controls, respectively. The representative image from the triplicate analysis is shown.

Given that the immortalized fibroblast cells in this study were sampled from different breeds and tissue types (ear, fetus, and lung), we analyzed the expression of three SLA class I genes, *SLA-1, SLA-2*, and *SLA-3*, and a class II gene, *SLA-DRB1*, using semiquantitative RT-PCR (Figure 4). Seven of the 10 cell lines, iKUF01, 04, 05, 06, 07, 08, and 09, expressed all three classical class I genes, though at varying levels. iKUF01 and iKUF05 showed higher *SLA-2* expression than the other cell lines. *SLA-1* expression was higher in iKUF04 and iKUF08 than in other cell lines. iKUF02 and iKUF10 lacked the expression of *SLA-3* and *SLA-1*, respectively. iKUF03 showed only a low level of *SLA-1* expression with no or undetectable levels of *SLA-2* and *SLA-3* expression. Interestingly, the levels and patterns of SLA class I gene expression varied among iKUF cell lines 01, 03, 04, 05, 06, 09, and 10 that were derived from the ear of different individuals, indicating, presumably, the presence of individual or haplotype-based differences in the expression of SLA class I genes. However, additional experiments should be conducted to prove this more clearly. In general, the expression of *SLA-3* was lower than that of *SLA-1* and *SLA-2*. The expression of *SLA-DRB1* was not observed in any of the cell lines, owing to the fact that fibroblasts do not express MHC class II molecules without cytokine stimulation (Umetsu et al., 1986; Kim et al., 2012).

### Increased Expression of SLA Class I Genes in Immortalized Fibroblast Cells

**FIGURE 5:**
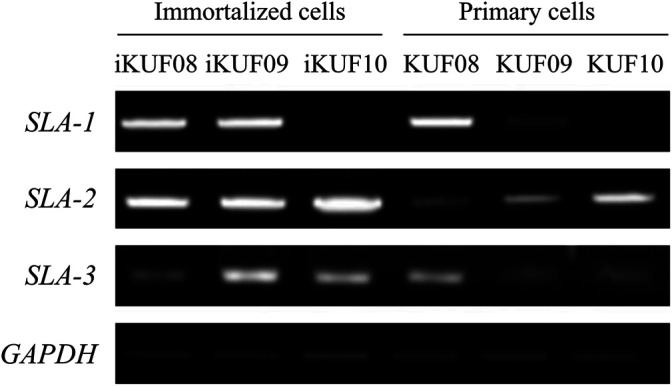
Comparison of SLA classical class I gene expression between primary and immortalized cells. Three immortalized cell lines, iKUF08, iKUF09, and iKUF10, are analyzed. KUF08, KUF09, and KUF10 are primary cells before immortalized. The names of the cells are on top. The expression of *SLA-1, -2*, and *-3* are being compared. *GAPDH* is used as a control. The representative image from the triplicate analysis is shown.

**FIGURE 6:**
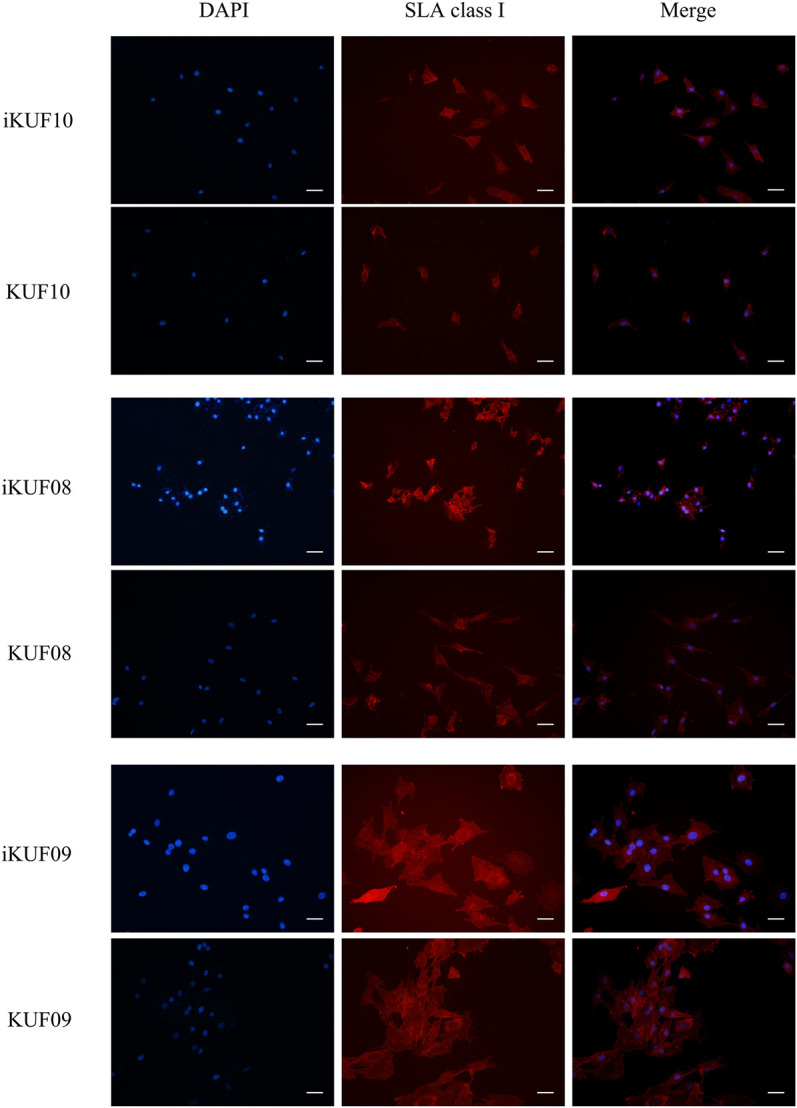
Immunohistochemical comparison of SLA classical class I gene expression between primary and immortalized cells. Three immortalized cell lines, iKUF10, iKUF08, and iKUF09 and corresponding primary cells, KUF10, KUF08 and KUF09 were used. The first and second columns show the results of DAPI staining and immunostaining by anti-pan SLA classical class I antibodies, respectively. The merged images of the two are shown in the third column. The first and second rows correspond to immortalized and primary cells, respectively. Scale bar: 50 µm. Magnification: × 20. The representative image from the triplicate analysis is shown.

We compared the expression levels of *SLA-1*, *-2*, and *-3* between iKUF08, iKUF09, and iKUF10 cells before and after immortalization (Figure 5). In both phases, all three genes were expressed in iKUF08 and iKUF09, but iKUF10 cells lacked *SLA-1* expression. The expression levels of SLA classical class I genes varied among the cell lines and were slightly upregulated in the immortalized compared to the primary cells based on semiquantitative reverse transcription polymerase chain reaction. The expression of SLA classical class I gene was also analyzed at the protein level by immunostaining with Pan-SLA classical class I-specific antibodies (Figure 6). Although the signal intensity of SLA-1 appeared to be consistent to that of the mRNA expression, clear conclusion on the protein expression level could not be drawn due to the quantitative limitations of immunohistochemical analysis.

## Discussion

Epstein–Barr virus-transformed B-lymphoblastoid cell lines and extensive curation have been important developments in the molecular characterization of MHC genes and MHC-antigen interactions related to HLA (Yang et al., 1989). Similar to B-lymphoblastoid cell lines, *hTERT*-transformed cells have extended cellular lifespans and are effectively used to prepare cells in sufficient number while maintaining their biological properties (Saldanha et al., 2003). In this study, we created a panel of 10 *hTERT*-transformed porcine fibroblast cell lines, conducted high resolution analysis of the major classical class I and class II SLA genes, including *SLA-1, SLA-2, SLA-3, SLA-DQB1, SLA-DRB1*, and *SLA-DQA*, and characterized the expression patterns of three class I genes and *DRB1* in each cell line. However, owing to the unavailability of MHC haplotype reference information, we were unable to reliably characterize the haplotypes of most of the six SLA genes in pigs. Together with other publicly available pig cell lines (Ho et al., 2009; Le et al., 2018), immortalized porcine cells could be used to establish a reference cell line panel for immunogenetic studies in pigs.

Except for nucleotide substitutions, structural variations in the SLA gene region, including *SLA-1* duplication in certain haplotypes, are not well studied in pigs (Le et al., 2020). To collectively improve our scientific understanding of haplotype diversity, gene duplication, pseudogenization, and conflicts in sequence assembly at the SLA region require systematic analysis using a common and reliable resource. Homozygous cell lines across the entire MHC region would be particularly useful. For HLA, the DNA fragments of the ∼5 Mbp MHC region of 95 MHC homozygous B-lymphoblastoid cell lines were captured and analyzed together with six alternative MHC reference sequences (Norman et al., 2017). In pigs, only three cell lines PK13, PK15, and 3D4/21 were reported as possibly homozygous across the entire SLA region, although the haplotype was identical for PK13 and PK15 (Ho et al., 2009; Le et al., 2018). Limited progress has been made with regard to porcine B-lymphoblastoid cell lines (Kaeffer, 1994).

The use of different typing methods between laboratories can contribute to heterogeneity in typing results (Erlich, 2012). The use of reference cell lines with diverse SLA haplotypes could resolve this artefact of methodological differences (Ho et al., 2009). Typing methods for SLA genes employing high throughput and parallel typing of multiple loci from genomic DNA have not been available. A gene panel of well characterized cell lines could greatly contribute to the development of the typing technology and the evaluation of its accuracy (Creary et al., 2019).

Understanding the kinetics of epitope binding to MHC molecules is important for the development of effective vaccines. However, resources for the systematic analysis of interactions between antigenic epitopes and SLA molecules are limited in pigs. We previously reported that the affinity of PCV2 ORF2 as an epitope to MHC class II molecules varied depending on the MHC haplotype (Le et al., 2018) and showed that the use of immortalized PAM cells can instead be used to evaluate the MHC binding affinity to antigenic peptides. The cell lines reported in this study could be applied in determining the binding affinity of epitopes to MHC class I proteins, a critical factor for CTL activation, without the complication of peptides binding to class II SLA molecules in the cell-based assay.

Our immortalized fibroblast cell lines showed lower growth rates than PK15 and did not form colonies under the anchorage-independent culture condition, suggesting greater similarity in phenotype to primary cells than available ATCC fibroblast cell lines. This is consistent with the results of a previous study on *hTERT* transfected porcine alveolar macrophages (Le et al., 2018). These characteristics may be more suitable in molecular biology studies that require primary fibroblast cells with relatively short passage numbers and showing lower accumulation of genetic mutations that could alter their original characteristics.

Resilience after pathogenic infection is likely to be affected by an individual’s immune capacity. Considering the functionality of SLA molecules, their expression should affect the consequence of adaptive immune responses and general animal health. The expression of MHC class I molecules varies depending on cell types and tissue origins (Fanbo et al., 2005). Our results showed variation in the expression level of SLA classical class I genes among the cell lines, independent of tissue type or individual breed. However, these results are only based on *in vitro* analysis. *In vivo* studies are required to describe the natural variation of SLA class I gene expression among individual pigs of different SLA haplotypes. Consistent to our observation, haplotype-specific expression patterns of SLA class I genes have been previously reported (Kita et al., 2012). Our fibroblast cell panel could be applied to investigate the regulatory mechanisms or genetic factors affecting the expression of SLA genes, which could inform animal breeding programs and improve the immune capacity within animal breeds. The cell lines also could be useful to study molecular biological mechanisms influencing MHC class I expression including autophagy and ER stress in pigs (Crotzer and Blum, 2009; Granados et al., 2009).

In this study, the expression levels of SLA class I genes were slightly higher in immortalized cells than in primary cells (Figure 5). Modo et al. (2003) found higher levels of MHC expression in the proliferative than in the non-proliferative or differentiating conditions in conditionally immortalized murine neural stem cells. Redondo et al. (1998) also showed that the expression level of MHC class II genes varied in lung tumor cell lines and observed a positive correlation between the expression of *HLA-DR* and proto-oncogene c-*myc*. The expression of MHC genes could be influenced by the rate of cell proliferation and may explain the increased expression of SLA class I genes in the immortalized fibroblast cell lines.

The development of diverse cell lines with various MHC haplotypes could be useful for immunogenetic analyses in pigs but the availability of these cell lines has been limited. Fibroblasts are relatively easy to isolate, culture, and immortalize compared to other livestock cell types and, therefore, appropriate to develop stable cell lines with confirmed MHC genotypes. Studies on variations in the genetic structures of SLA and their expression using reference cell lines have great potential to enhance our knowledge regarding the adaptive capacity and regulatory elements of the porcine immune system. Although MHC genes of mammalian species are evolutionary conserved, the organization of the MHC region in pigs differs from others in that MHC class II and I genes are separated by a centromere (Lunney et al., 2009). The immortalized fibroblast cell lines in this study also could be useful to understand the influence of the centromere on generating haplotype diversity of the MHC region through genetic recombination.

## Data Availability

The datasets presented in this study can be found in online repositories. The names of the repository/repositories and accession number(s) can be found in the article/Supplementary Material.
