# Supplementary material for: Development of an Immortalized Porcine Fibroblast Cell Panel With Different Swine Leukocyte Antigen Genotypes
Source: Front Genet. 2022 Feb 7;13:815328. doi: 10.3389/fgene.2022.815328 (PMC8859410; doi:10.3389/fgene.2022.815328)
Supplement: Supplementary file 1 [file Table1.docx]

Supplementary Table 1. Primer information used in this study

| Target gene | Primer name | Sequence (5'–3') | Target region | Use | Annealing temperature | Amplicon size (bp) |
| --- | --- | --- | --- | --- | --- | --- |
| SLA-1 | SLA1-e1F1^a^ | MTAARCTCTCCRCCCASCCGGCTCTG | Exons 2 and 3 | Genotyping and semi-quantitative PCR | 65℃ | 1844 |
|  | SLA-e4R4 | cGGGTCACATGTGTCyTTGGAGG |  |  |  |  |
|  | SLA1-Seq. 2-F | tgctatgctgtgCGCCGARAGGAGGGT | Exon 2 | Sequencing | 50℃ |  |
|  | SLA1-Seq. 3-F | atgctgattatcgCCCKGGTTGGWCGCG | Exon 3 |  |  |  |
| SLA-2 | SLA2-e1F^b^ | GCCTCGACACAGAATCTCCGatataTCCAAAGATG | Exons 2 and 3 | Genotyping | 65℃ | 1751 |
|  | SLA-e4R4 | cGGGTCACATGTGTCyTTGGAGG |  |  |  |  |
|  | SLA2-e1F | GCCATGCTCATTCTGCTGTC |  | semi-quantitative PCR | 61℃ | 649 |
|  | SLA-e4R4 | CGGGTCACATGTGTCYTTGGAGG |  |  |  |  |
|  | SLA-i1F | tgctatgctgtgCGCCGARAGGAGGGT | Exon 2 | Sequencing | 50℃ |  |
|  | SLA-i2F | atgctgattatcgCCCKGGTTGGWCGCG | Exon 3 |  |  |  |
| SLA-3 | SLA3-spF5 | GGGGrCCCTGGCCCTGAtT | Exons 2 and 3 | Genotyping | 65℃ | 1655 |
|  | SLA-e4R4 | cGGGTCACATGTGTCyTTGGAGG |  |  |  |  |
|  | SLA1/3f#92^c^ | CCAGACTCCGAGGCTGAGGAT |  | semi-quantitative PCR | 50℃ | 1494 |
|  | SLA3r#121 | TAGGCTCTTTTCCCTTGGTTAGG |  |  |  |  |
|  | SLA3-seq2-F6 | CTGTGAATGCTGCTGCGCCGAGAGGAGGGT | Exon 2 | Sequencing | 50℃ |  |
|  | SLA3-seq2-R7 | AAKGCCSGGGTGACCCT |  |  |  |  |
|  | SLA3-seq3-F4 | TGTGAATGCTATGGTKGGTCGYGGC | Exon 3 |  |  |  |
|  | SLA3-seq3-R5 | GACCCCCTTTTCCTCT |  |  |  |  |
| DQA | DQAi1F3 | CTAGAGACTGTGCCACAGATGAAG | Exon 2 | Genotyping | 65℃ | 898 |
|  | DQAe3R1 | ACAGATGAGGGTGTTGGGCTGA |  |  |  |  |
|  | DQAi1F4 | GTMAAGTTCTCTTGTCAC |  | Sequencing | 50℃ |  |
| DQB1 | DQB1F-119 | GCGGCGGGTTTCAGGTGGATG |  | Genotyping | 65℃ | 478 |
|  | DQB1R | aaccctcactaaagACCCACTCTCTCYGCGCGGWGTCTC |  |  |  |  |
|  | Sq-mul-DQB1 | AACCCTCACTAAAG |  | Sequencing | 50℃ |  |
| DRB1 | DRB1F-22 | gaatgctgcgactacctgTGGATCATTGCTGTCCACGCAGMG |  | Genotyping | 65℃ | 364 |
|  | DRB1R + 284 | tctaccaggcattcgcttcatiiiiiCYSCSGGCVGCSCA |  |  |  |  |
|  | CpDRB5a^d^ | TCTGTGTTTCTCCAGAGGCT |  | semi-quantitative PCR | 63℃ | 760 |
|  | CpDRB3b | GCGTCCTTTCTGATTCTTGAAGTA |  |  |  |  |
|  | Sq-mul-DRB1 | TAGCTGAATTCGAATGCTGCGACTA |  | Sequencing | 50℃ |  |
| Vimentin | Vimentin-F^e^ | TGTCCGCCAGCAGTATGA | Exon 4 | semi-quantitative PCR | 58℃ | 233 |
|  | Vimentin-R | CTTCCATTTCCCGCATCT |  |  |  |  |
| GAPDH | GAPDH-SP-F | GCTACACTGAGGACCAGGTTG | Exon 6 | semi-quantitative PCR | 58℃ | 294 |
|  | GAPDH-SP-R | AGGAGATGCTCGGTGTGTTG |  |  |  |  |

^a^ Le et al. (2020)

^b^ Choi et al. (2015)

^c^ Ho et al. (2006)

^d^ Thong et al. (2011)

^e^ Shi et al. (2013)
