# Supplementary material for: Development of an Immortalized Porcine Fibroblast Cell Panel With Different Swine Leukocyte Antigen Genotypes
Source: Front Genet. 2022 Feb 7;13:815328. doi: 10.3389/fgene.2022.815328 (PMC8859410; doi:10.3389/fgene.2022.815328)
Supplement: Supplementary file 2 [file DataSheet1.DOCX]

**
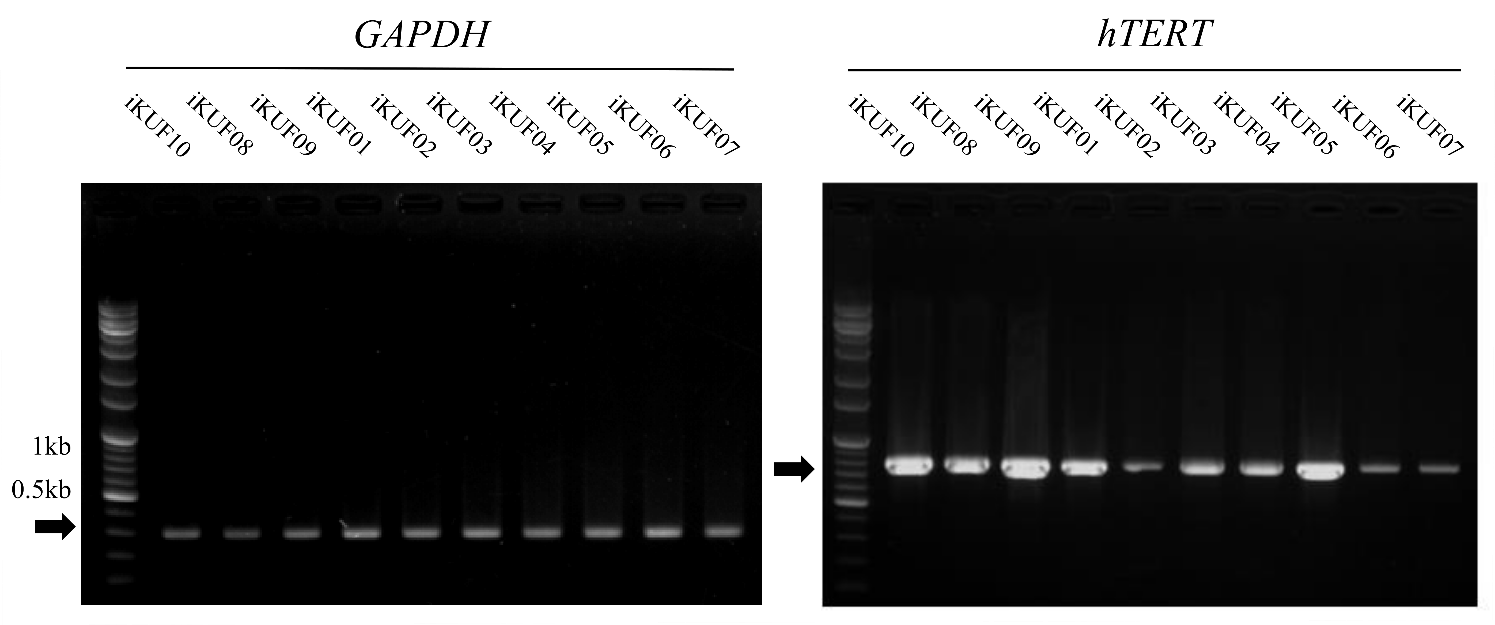
**

**Supplementary Figure 1.** Expression analysis of *hTERT* using reverse transcription PCR for 10 immortalized porcine fibroblast cells. The names of the cell lines are indicated on top. *GAPDH* is used as a control for RT-PCR. Product sizes are indicated by the arrows, *GAPDH* was 296 bp and *hTERT* was 778 bp.
